# Emergency department use by patients who received chimeric antigen receptor T cell infusion therapy

**DOI:** 10.3389/fonc.2023.1122329

**Published:** 2023-03-17

**Authors:** Demis N. Lipe, Aiham Qdaisat, Patrick Chaftari, Monica K. Wattana, Pavitra P. Krishnamani, Cielito Reyes-Gibby, Sai-Ching J. Yeung

**Affiliations:** ^1^ Department of Medical Services, IQVIA Biotech, Houston, TX, United States; ^2^ Department of Emergency Medicine, The University of Texas MD Anderson Cancer Center, Houston, TX, United States

**Keywords:** chimeric antigen receptor T cells, emergency department, CAR T cells, utilization, Mortality, disposition, length of stay, oncologic emergency

## Abstract

**Background:**

Chimeric antigen receptor T cell infusion (CAR T) therapy has revolutionized the treatment of hematologic malignancies, but treatment-related toxicities are of concern. Understanding the timing and reasons for which patients present to the emergency department (ED) after CAR T therapy can assist with the early recognition and management of toxicities.

**Methods:**

A retrospective observational cohort study was conducted for patients who had undergone CAR T therapy in the past 6 months and visited the ED of The University of Texas MD Anderson Cancer Center between 04/01/2018 and 08/01/2022. The timing of presentation after CAR T product infusion, patient characteristics, and outcomes of the ED visit were examined. Survival analyses were conducted using Cox proportional hazards regression and Kaplan-Meier estimates.

**Results:**

During the period studied, there were 276 ED visits by 168 unique patients. Most patients had diffuse large B-cell lymphoma (103/168; 61.3%), multiple myeloma (21/168; 12.5%), or mantle cell lymphoma (16/168; 9.5%). Almost all 276 visits required urgent (60.5%) or emergent (37.7%) care, and 73.5% of visits led to admission to the hospital or observation unit. Fever was the most frequent presenting complaint, reported in 19.6% of the visits. The 30-day and 90-day mortality rates after the index ED visits were 17.0% and 32.2%, respectively. Patients who had their first ED visit >14 days after CAR T product infusion had significantly worse overall survival (multivariable hazard ratio 3.27; 95% confidence interval 1.29–8.27; P=0.012) than patients who first visited the ED within 14 days of CAR T product infusion.

**Conclusion:**

Cancer patients who receive CAR T therapy commonly visit the ED, and most are admitted and/or require urgent or emergent care. During early ED visits patients mainly present with constitutional symptoms such as fever and fatigue, and these early visits are associated with better overall survival.

## Introduction

Since the advent of chimeric antigen receptor T cell infusion (CAR T) therapy for hematologic malignancies, much has been learned and recognized about the risks and complications associated with CAR T therapy. Toxicities such as cytokine release syndrome (CRS), immune effector cell-associated neurotoxicity syndrome (ICANS), and infections in the days to months following the infusion of CAR T products have been recognized. The incidence rates of CRS and ICANS have been reported in the literature to range from 57% to 93% and 20% to 70%, respectively (1–3). These toxicities vary greatly and are thought to be influenced by multiple factors, including patient characteristics, tumor burden, CAR T cell dose, and differences in manufacturing processes, among others (4). The current treatment strategy of associated toxicities is focused on reduction of the overall inflammation by use of corticosteroids or cytokine inhibition, which is based on the grading of the toxicity as defined by the American Society for Transplantation and Cellular Therapy (ASTCT) guidelines on the management of CAR T related toxicities (5).

Although much is known about how clinicians should recognize, work up, and manage these toxicities (1), there is insufficient published literature on the use of the emergency department (ED) by this cohort of patients. CAR T therapy recipients are generally believed to have a potential for increased health care use after CAR T therapy, with high rates of intensive care unit (ICU) admissions and prolonged lengths of stay in the hospital, although re-hospitalization patterns appear to vary based on whether the patient’s CAR T therapy was an inpatient or outpatient event (6, 7). One study reported that hospital re-admission and ICU admission rates within the first 3 months after CAR T product infusion were 28.1% and 15.5%, respectively (7). Another study reported that nearly 40% were re-hospitalized and 21% visited the ED during the initial 12 months following CAR T product infusion (8). Reasons for re-hospitalizations or ED visits have been mostly related to the primary disease, pain, CAR T- related toxicities, and infection (6–8). However, despite these reports, much is still unknown, and more data is needed to better understand why patients who receive CAR T therapy visit the ED and what their outcomes are.

In the current study, we describe the reasons why patients at a single comprehensive cancer center visited the ED after CAR T product infusion, as well as their outcomes, including disposition (whether the patient was discharged, admitted, transferred, or other), hospitalization, and survival.

## Methods

### Population

A retrospective observational cohort study was conducted by identifying all cancer patients who visited the ED of The University of Texas MD Anderson Cancer Center (a comprehensive cancer center in Houston, Texas, USA) within 6 months after receiving any CAR T product infusion, using the institutional data warehouse. For each patient with multiple ED visits, the first ED visit after CAR T product infusion was identified as the index ED visit. The period studied was between 04/01/2018 and 08/31/2022 for the index ED visits. Patients who were less than 18 years of age at their index ED visit were excluded.

### Study setting

Our institution is a comprehensive cancer center that established the first academic emergency medicine department in 2010. The ED is staffed by board certified emergency and internal medicine physicians and has 44 beds, serving approximately 26,000 patients annually. The patients that visit the ED are assessed and treated by the staff in the ED, in consultation with the patient’s oncologist. There is also an ED-run observation unit in the hospital, which serves patients projected to need in-hospital care for less than 2 midnights. This unit is functional 24-hours a day and is staffed by an emergency or internal medicine physician along with advanced practice providers. Most patients who are placed in the observation unit originate from the ED; however, patients may also come directly from clinics or procedure areas (9). Additionally, patients may also be admitted directly to the hospital by their oncologists, while bypassing the ED.

### Variables and data collection

Thirty-day and 90-day mortality rates for the ED visits were calculated from the time of the index ED presentation to the reported time of death. ICU admission was reported as any ICU admission during the patient’s hospital stay associated with the index ED visit. In-hospital mortality was identified as a death during the ED visit or subsequently during the hospital admission associated with the index ED visit. The timing of the indexed ED visit was grouped based on the time from CAR T product infusion to the ED presentation, and was categorized as early (≤14 days after CAR T product infusion) or late (>14 days after CAR T infusion). CAR T product infusion was defined as the day the CAR T product was infused. Because the first 14 days after CAR T infusion are the most critical with regards to treatment-related toxicities (10, 11), we chose the time point of 14 days as the cut-off to define early versus late presentation. The acuity level assigned to the patient was based on the modified Emergency Severity Index (mESI) tool used to triage patients in our ED. Life-threatening is level 1, emergent level 2, urgent level 3, less urgent level 4, and non-urgent level 5. This five-level triage algorithm classifies patients based on disease severity at presentation and the expected resource utilization (12). The “presenting complaint” was defined as the patient’s reported reason for visiting the ED at the time of the triage assessment.

Clinical and demographic information, ED visit-related data, and outcomes were collected from patients’ electronic health records and the institution’s data warehouse. Race and ethnicity groups were categorized according to the Office of Management and Budget standards for race and ethnicity (13). The diagnosis of CRS, ICANS, or active infection(s) was collected by reviewing the physician(s) and ED visit notes, reporting the grade for the CRS and the ICANS at the time of the ED visit if present.

### Statistical analysis

Patient-level and visit-level data were reported using descriptive statistics. Medians and interquartile ranges were reported for continuous variables. Numerical data were evaluated for normality using quantile-quantile plots, histogram plots, and the Shapiro-Wilk test. Categorical variables were reported as counts and percentages. Statistical significance was appraised for proportions of categorical variables using the chi-square test or the Fisher exact test, as indicated. The Wilcoxon-Mann-Whitney test was used to determine significant differences for continuous variables (all data were not normally distributed).

For the survival analysis, survival time was defined as the time interval from the date of CAR T product administration onto the date of death or the end of the observation period, censoring patients who were lost to follow-up on the dates of their last recorded clinic visit or communication (email, video conference or phone call). We used the Kaplan-Meier method followed by the log-rank test to assess differences in overall survival between patients with early and late ED presentations. Univariate and multivariable Cox proportional hazards regression models were used to assess the association between different clinical factors and overall survival, reporting the hazard ratio and its 95% confidence interval. For the final model, the proportional hazards, the non-linearity, and the influential observations assumptions were evaluated by examining the Schoenfeld residuals, the Martingale residuals, and the Deviance residuals.

All statistical analyses were performed using R software (version 4.0.3, The R Foundation, http://www.r-project.org). Two-sided P values less than 0.05 were considered statistically significant.

### Ethics

The Institutional Review Board of The University of Texas MD Anderson Cancer Center approved the study and granted a waiver of informed consent.

## Results

### Patient characteristics

**Figure 1:**
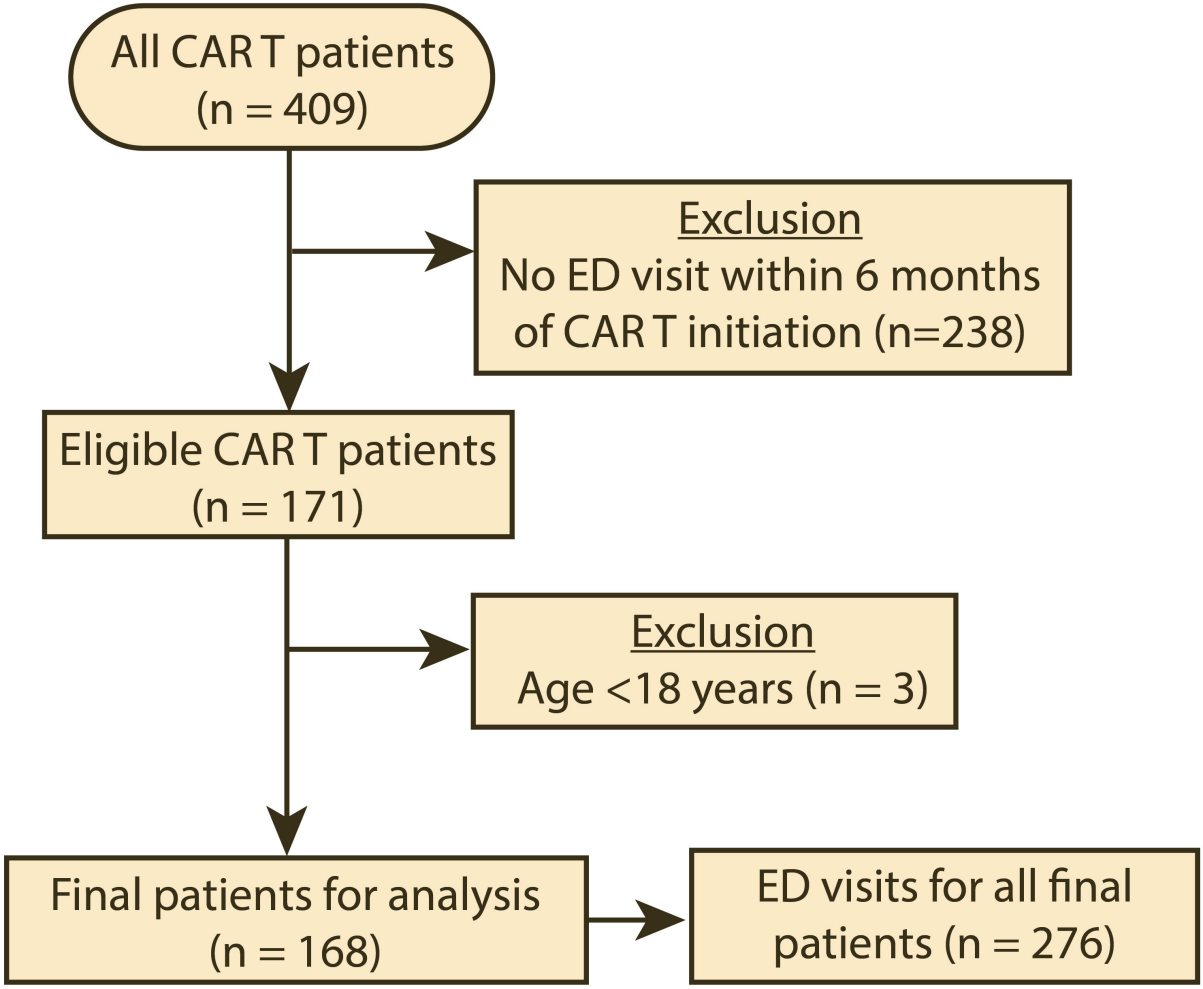
Flow diagram of the cohort selection for the period studied (04/01/2018–08/31/2022). CAR T, chimeric antigen receptor T-cell infusion therapy; ED, emergency department.

During the period studied, 409 patients received CAR T therapy, and 171 (41.8%) had at least one ED visit within 6 months of CAR T product infusion (**Figure 1**). The clinical and demographic characteristics of the patients included in the analysis (n=168) are summarized in **Table 1**. The median age for the patients in our cohort was 63 years (interquartile range: 54–69). Most were male (60.7%), white (70.8%), and not of Hispanic or Latino ethnicity (72.6%). The most common cancer types for which CAR T therapy was initiated were diffuse large B-cell lymphoma (61.3%), multiple myeloma (12.5%), mantle cell lymphoma (9.5%), and acute lymphoblastic leukemia (6.0%). Eighteen patients (10.7%) had other cancer types. During the period studied, the median number of ED visits was 1 (interquartile range 1–2). For the type of CAR T product, 108 patients (64.3%) were treated with axicabtagene ciloleucel within 6 months prior to their ED visit (**Table S1**). The remaining ED visits were by patients who were treated with idecabtagene vicleucel (12.5%), brexucabtagene autoleucel (11.3%), tisagenlecleucel (7.1%), and lisocabtagene maraleucel (4.8%). All the patients had their CAR T product infusion administered in an inpatient setting. **Table S3** summarizes the presentation and characteristics of the ED visits stratified by CAR T product type, while **Table S4** summarizes the presentation and characteristics of the ED visits stratified by the underlying cancer type.

**Table 1:** Clinical and demographic characteristics of patients presenting to the emergency department (ED) after chimeric antigen receptor T-cell infusion therapy (n = 168).

Characteristic	No. (%)
Age, median (IQR), years	63 (54–69)
Sex	
Female	66 (39.3)
Male	102 (60.7)
Race	
White	119 (70.8)
Black or African American	14 (8.3)
Asian	9 (5.4)
Others	23 (13.7)
Unknown or declined to answer	3 (1.8)
Ethnicity	
Not Hispanic or Latino	122 (72.6)
Hispanic or Latino	37 (22.0)
Unknown or declined to answer	9 (5.4)
Charlson comorbidity index, median (IQR)	4 (3–5)
Cancer type	
Diffuse large B-cell lymphoma	103 (61.3)
Multiple myeloma	21 (12.5)
Mantle cell lymphoma	16 (9.5)
Acute lymphoblastic leukemia	10 (6.0)
Others	18 (10.7)
Number of ED visits, median (IQR)	1 (1–2)

IQR, interquartile range.

### ED visits and outcomes

During the period studied, patients included in our analysis made 276 unique visits to our ED. Most of the visits were of high acuity; 167 (60.5%) were urgent, and 104 (37.7%) were emergent or life-threatening. Only 5 visits (1.8%) had an acuity level of “less urgent” or “non-urgent”. In terms of presenting complaints, around one-fifth of the visits (19.6%) were for a fever, and fatigue was reported in 9.1% of the visits. Altered mental status was reported in 5.1% of the visits, hypotension in 4.7%, and suspected sepsis in 4.3%. Abdominal pain (6.5%), shortness of breath (6.2%), cough (5.4%), nausea and/or vomiting (4.3%), and dizziness (1.8%) were also commonly reported as a presenting complaint. Other complaints are reported in **Table 2**. CRS was reported in 21 patients (7.6%) at the time of the ED visit, with the majority (19/21) being grade 1, while ICANS was reported in only 9 ED visits, 4 of which were higher than grade 1 (**Table 2**). Tocilizumab and corticosteroids were administered during the ED stay in 1.1% and 5.8% of the visits, respectively (**Table 3**). In addition, infection was identified in 81 (29.3%) visits (**Table 2**), of which 18.5% (15/81) tested positive for COVID-19 at the ED visit. Within 14 days before the ED visit, COVID-19 was reported in 17 (6.2%) of the visits, including the aforementioned 15 (5.4%) active cases. Fever (≥38°C) was recorded in 9.8% of the ED visits. Antibiotics were administered in the ED in 50.7% of the visits (**Table 3**). Of significance, infections were higher in very late (>90 days after CAR T product infusion) visits compared to the early (≤14 days) visits (36.2% vs.13.8%, respectively; **Table S2**). Patients presented with severe neutropenia (<0.5 x 10^9/L) in 12.0% of the visits; while 48.6% of the ED visits were associated with severe thrombocytopenia (<50 x 10^9/L; **Table 2**).

**Table 2:** Characteristics of emergency department visits by cancer patients in our analysis who had initiated chimeric antigen receptor T-cell infusion therapy within the past 6 months (total number of visits = 276).

Characteristic	No. (%)
Acuity	
Urgent	167 (60.5)
Emergent	104 (37.7)
Less urgent	4 (1.4)
Non-urgent	1 (0.4)
Top presenting complaints*	
Fever	54 (19.6)
Abnormal lab results	28 (10.1)
Fatigue	25 (9.1)
Abdominal pain	18 (6.5)
Shortness of breath	17 (6.2)
Cough	15 (5.4)
Altered mental status	14 (5.1)
Hypotension	13 (4.7)
Suspected sepsis	12 (4.3)
Fall	12 (4.3)
Nausea and/or vomiting	12 (4.3)
Diarrhea	7 (2.5)
Dizziness	5 (1.8)
Chest pain	4 (1.4)
Extremity weakness	3 (1.1)
Constipation	3 (1.1)
Leg swelling	3 (1.1)
Other pain	14 (5.1)
CRS	
No	255 (92.4)
Grade 1	19 (6.9)
Grade 2	2 (0.7)
ICANS	
No	267 (96.7)
Grade 1	5 (1.8)
Grade 2	2 (0.7)
Grade 3	2 (0.7)
Identified infection	
No	195 (70.7)
Yes	81 (29.3)
Temperature at presentation, median (IQR), °C	36.9 (36.6, 37.3)
Fever (≥38°C) recorderd in the ED	
No	249 (90.2)
Yes	27 (9.8)
WBC count, median (IQR) × 10^9^/L	3.0 (1.7, 4.8)
Severe neutropenia (< 0.5 x10^9^/L)	
No	243 (88.0)
Yes	33 (12.0)
Severe thrombocytopenia (<50 x 10^9/L)	
No	142 (51.4)
Yes	134 (48.6)
COVID-19 within 14 days of the ED visit	
No	259 (93.8)
Yes	17 (6.2)

CRS, cytokine release syndrome; ICANS, immune effector cell-associated neurotoxicity syndrome; °C, Celsius.

*Only complaints occurring in more than 1% of the visits were reported. In some visits, the patient presented with more than one complaint.

**Table 3:** Outcomes of emergency department (ED) visits for cancer patients in our analysis who had received chimeric antigen receptor T-cell infusion therapy within the past 6 months (total number of visits = 276).

Characteristic	No. (%)
ED disposition	
Admit	169 (61.2)
Discharge	68 (24.6)
Observation	34 (12.3)
Others*	5 (1.8)
ED median length of stay (IQR), hours	7 (5–9)
ICU admission	
No	262 (94.9)
Yes	14 (5.1)
Administration of antibiotics during ED stay	
No	136 (49.3)
Yes	140 (50.7)
Administration of tocilizumab during ED stay	
No	273 (98.9)
Yes	3 (1.1)
Administration of corticosteroids during ED stay	
No	260 (94.2)
Yes	16 (5.8)
Hospital median length of stay^†^ (IQR), days	6 (4–9)
Death during the ED visit or subsequent hospital admission	
No	262 (94.9)
Yes	14 (5.1)
Death within 30 days of ED visit	
No	229 (83.0)
Yes	47 (17.0)
Death within 90 days of ED visit	
No	187 (67.8)
Yes	89 (32.2)

IQR, interquartile range; ICU, intensive care unit.

*Includes visits in which the patient left without being seen (n = 2), was transferred (n = 2), or left against medical advice (n = 1).

†Includes only visits in which the patient was admitted (n = 169).

As for the ED visit outcomes, 169 visits (61.2%) resulted in the patient being admitted to the hospital, and 68 (24.6%) resulted in discharge home (**Table 3**). Thirty-four visits resulted in the patient being placed in the observation unit. For patients who were admitted to the hospital, the median hospital length of stay was 6 days (interquartile range 4–9). When stratified by timing of the ED visit, visits that occurred within 14 days of CAR T product infusion had significantly higher rates of fever and fatigue as presenting complaints than those that occurred more than 90 days after CAR T product infusion (fever: 41.4% compared with 19.1%, P = 0.006; fatigue: 13.8% compared with 3.2%, P = 0.030; **Table S2**). For almost one-third of the visits (32.2%), the patient died within 90 days of the ED visit (**Table 3**). The thirty-day mortality rate for these ED visits was 17.0%.

### Overall survival

**Figure 2:**
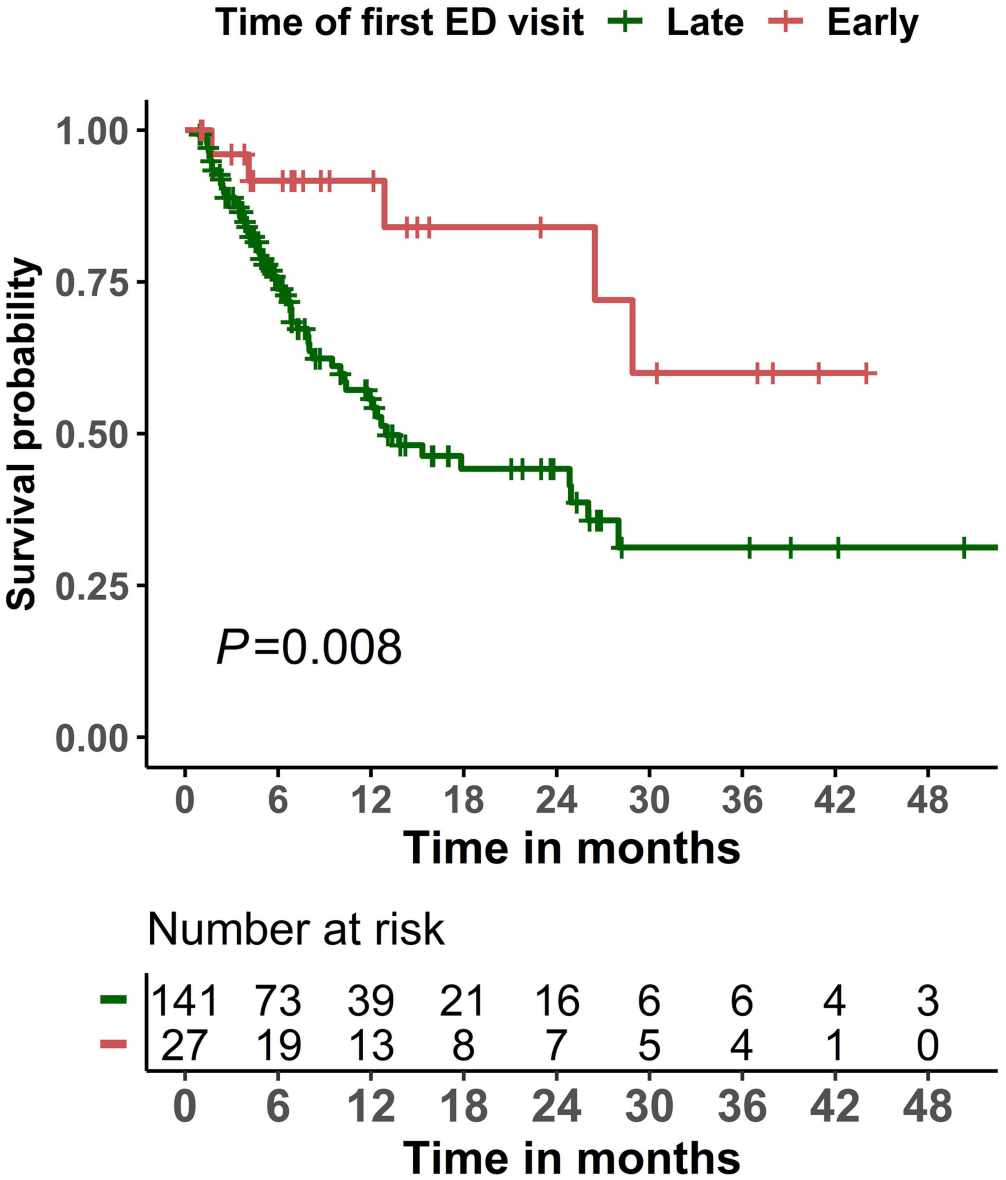
Kaplan-Meier overall survival curves among patients who visited the emergency department (ED) within 6 months of initiating chimeric antigen receptor T-cell infusion therapy, stratified by timing of the first ED visit (early: ≤14 days; late: >14 days).

The mortality rate during the study period was 37.5% (63/168). The cause and place of death are summarized in **Table S5**. Cancer progression (17.5%), infection/sepsis (14.3%), and organ failure (17.5%) were the most frequent causes of death in our cohort. Twenty-seven (42.9%) patients had their cause of death reported as unknown or not documented in the medical records. Only 42.9% deaths occurred in hospitals. For the subset of patients who died during the ED visit or subsequent hospital admission, infection was the most common cause of death (42.9%) for these patients (**Table S6**). Patients whose first ED visit occurred early (within 14 days of CAR T product infusion) had significantly (P=0.008) better overall survival than those whose first ED visit occurred late (more than 14 days after product infusion; **Figure 2**). Similar results were observed when a two-year survival was examined (**Figure S1**). As for the Cox regression analyses, non-linearity was detected for age and the Charlson comorbidity index, and we therefore categorized these variables into two groups. When compared with an early first ED visit (reference), patients whose first ED visit occurred >14 days after CAR T product infusion had significantly worse overall survival in both the univariate Cox regression analysis (hazard ratio 3.23, 95% confidence interval 1.29–8.10, P = 0.013) and the multivariable Cox regression analysis (hazard ratio 3.27, 95% confidence interval 1.29–8.27, P = 0.012; **Table 4**).

**Table 4:** Univariate and multivariable Cox proportional hazards analyses of overall survival in cancer patients who visited the emergency department within 6 months after CAR T product infusion (n = 168).

Variable	Univariate		Multivariable	
	HR (95% CI)	P	HR (95% CI)	P
Age				
<65 years	Reference			
≥65 years	1.18 (0.71–1.96)	0.516	1.17 (0.69–1.98)	0.571
Sex				
Female	Reference			
Male	1.33 (0.79–2.25)	0.283	–	–
Race				
Non-White	Reference			
White	1.16 (0.67–2.01)	0.594	–	–
Charlson comorbidity index				
≤2	Reference			
>2	1.00 (0.52–1.89)	0.990	0.90 (0.47–1.72)	0.742
Main cancer type				
Multiple myeloma	Reference			
Leukemia	1.33 (0.40–4.38)	0.641	1.38 (0.39–4.82)	0.617
Lymphoma	1.20 (0.47–3.02)	0.702	1.33 (0.52–3.42)	0.549
Time to first ED visit				
Early (≤14 days after product infusion)	Reference			
Late (>14 days after product infusion)	**3.23 (1.29–8.10)**	**0.013**	**3.27 (1.29–8.27)**	**0.012**

HR, hazard ratio; CI, confidence interval; ED, emergency department. Boldface indicates P < 0.05.

## Discussion

Integrating CAR T therapy into the treatment of hematologic malignancies paved the way for better survival outcomes (14–16). However, as with every other cancer therapy, adverse events are a concern. In the current study, we examined the reasons for ED use by patients who received CAR T therapy and their outcomes. We found that when patients visited the ED after CAR T therapy, they mainly complained of constitutional symptoms, including fever and fatigue, and for most of these visits (73.5%), the patient was admitted to either the hospital or placed in the observation unit. It has been previously reported that the presence of fever does not seem to affect the safety and efficacy of CAR T therapy, however, the same study suggested that the absence of fever indicates a poor response to CAR T therapy (17). Although 19.6% of the presenting complaints included fever, an infectious etiology for the visit was found in over 29% of the cases. Of these cases, 5.4% were due to COVID-19; however, only two patients died as a direct cause of COVID-19, and 80.0% were admitted. While the outcomes of COVID-19 in patients treated with CAR T therapy remain unclear, one study reported a prevalence of COVID-19 of 4.8% and a mortality rate of nearly 50% in patients who had received CAR T therapy (18). This is likely due to the immunocompromised state after CAR T product infusion but is also affected by other factors, such as malignant disease state and comorbidities. ED clinicians must recognize that these patients have a much higher rate of complications from COVID-19 and should have a lower threshold for admitting them.

Our study shows that of patients presenting to ED within 6 months of CAR T cell therapy, CRS and ICANS was present in 7.6% and 3.3%, respectively. This is likely because the CAR T product administration in all our patients was done in an inpatient setting, during which these toxicities were closely monitored and treated during their inpatient hospital stay. The CRS and ICANS diagnosed at their presentation to our ED were those cases with delayed or late occurrence. In our cohort, most of the CRS and the ICANS reported during the ED visit were of grades 1 or 2. Our institutional guidance recommends patients stay within 30 minutes of the hospital for 30 days after CAR T product infusion, therefore this might have prompted patients to present earlier in the course of their illness, however, as CAR T product administration is increasingly being done as an outpatient procedure, ED physicians need to be aware of these toxicities, including the optimal evaluation, grading, and management plans (1), as higher grades (grade 3 and grade 4) are to be expected with the shift to outpatient administration.

As for the timing of the ED visits, those that occurred within 14 days of CAR T product infusion had significantly higher rates of fever and/or fatigue as a presenting complaint, with CRS and ICANS reported in these visits. Patients who first visited the ED early after CAR T product infusion had better overall survival outcomes compared with those who initially visited the ED later. After CAR T therapy, some patients present to the ED for the management of inflammatory events associated with the CAR T product, with certain severe cases needing to be admitted to the ICU (8, 19, 20). Our study showed that only a minority of the ED visits (5.1%) resulted in an ICU admission. However, 26.2% of the patients that presented to the ED had an ICU admission at some point within three months after CAR T therapy, either through direct admission or transfer to the ICU from initial hospitalization for CAR T-cell infusion. While the ICU stays resulting from ED visits have not been explored before, the overall ICU admission rate is similar to a recent international multicenter report showing that up to 27% of patients required ICU admission after CAR T therapy. Other studies reported different ICU admission rates ranging from 10-47% (7, 19, 21). The overall differences in ICU admission rates may stem from differences in local practices, especially the use of in-hospital infusion versus outpatient infusion. In this study, all the patients had in-hospital CAR T therapy infusion; therefore, CAR T therapy toxicities were closely monitored during that patient’s inpatient stay prior to their ED presentation and were admitted to the ICU during that stay if needed. Additionally, our institutional guidance recommends patients stay within 30 minutes of the hospital for 30 days after CAR T product infusion; therefore, this might have prompted patients to present early in the course of their illness. Only 1 (1.6%) patient had CAR T therapy toxicity as the main cause of death.

For patients who first visited the ED later (>14 days of CAR T product infusion), in whom we observed worse overall survival outcomes, it is known that cancer patients frequently visit the ED near the end of life, with reasons mainly related to cancer progression (22). Moreover, and as previously reported, post CAR T relapse can be observed early after infusion and is associated with poor overall survival, mainly due to the persistence or progression of the primary malignant disease (23, 24). In our study, cancer progression was a main cause of death especially for patients who died later after their ED visit and/or the subsequent hospital admission, suggesting the utilization of ED by these patients near the end of life and explaining the poor survival outcomes for these patients. However, the interpretation of these results should pay heed to the fact that all the patients in this study had at least one ED visit, and other patients who had CAR T infusions but never visited the ED after their treatment may have other characteristics and different survival outcomes.

Mortality rates after the ED visits in our study were high, with a 30-day mortality rate of 17.0% and a 90-day mortality rate of 32.2%. To the best of our knowledge, the current study is the first to evaluate mortality rates in patients who visited the ED after CAR T therapy. A systematic review reported that fatal toxic side effects may occur with up to a 5% mortality rate within the first 30 days of CAR T therapy, but the risk of death varied depending on the product administered and other disease-specific factors (25). Additionally, others have reported non-relapse mortality associated with CAR T therapy to be as high as 15% overall, with infections and neurologic toxicities being major contributors to mortality (26). For this reason, it is important that ED clinicians are able to recognize these toxicities quickly when patients present to the ED after CAR T therapy.

Certain limitations accompanied our study, mainly due to the retrospective nature of the study. First, our cohort consisted of only patients who presented to the ED within 6 months of initiating CAR T therapy. The characteristics of patients who did not come to the ED could be different and need to be further investigated. In this study, interpretation of the reported toxicities needs to take into consideration that the prevalence rates reported in our study are limited to those reported at the time of the ED visit, which happened after the patients were discharged from their inpatient stay for CAR T product infusion, where we anticipate most of the toxicities occurred. Second, in the current study, we reported specifically the reasons for presentation to the ED. Such an approach limited the integration of important clinical variables during pre- or post-ED visits, including adverse events that were presented elsewhere or events that occurred before or after the ED visit. Similarly, we could have possibly missed patients who presented to another ED or were admitted directly to the hospital. Finally, the cause of death was unknown or undocumented in 42.9% of the patients, for which a prospective study is needed to have a better and a complete understanding of the causes of death in these patients.

In conclusion, we found that cancer patients who receive CAR T therapy commonly visit the ED, and the timing of the visit is associated with different survival outcomes in these patients. Early ED visits appear to be related to the early systemic inflammatory response resulting from CAR T therapy and are associated with better overall survival. Most of the patients were admitted, and the 90-day mortality rate after these visits was high (32.2%). Additional future studies are needed to further investigate and identify characteristics of early presentation to the ED that can be used as a predictor of response to the treatment.

## Data availability statement

The original contributions presented in the study are included in the article/**Supplementary Material**. Further inquiries can be directed to the corresponding author.

## Ethics statement

The studies involving human participants were reviewed and approved by The University of Texas MD Anderson Cancer Center. Written informed consent for participation was not required for this study in accordance with the national legislation and the institutional requirements.

## Author contributions

DL, AQ, and S-CY conceived and designed the study and developed the methods. AQ and PC collected the data. AQ was responsible for content analysis. S-CY supervised the statistical analysis. DL and AQ created the figures and tables. DL, AQ, PC, MW, PK, CR-G, and S-CY interpreted the results and drafted the first version of the manuscript. All authors contributed to the article and approved the submitted version.

## References

[1] Santomasso BD Nastoupil LJ Adkins S Lacchetti C Schneider BJ Anadkat M. Management of immune-related adverse events in patients treated with chimeric antigen receptor T-cell therapy: Asco guideline. J Clin Oncol (2021) 39(35):3978–92. doi: 10.1200/JCO.21.01992 34724386

[2] Sheth VS Gauthier J. Taming the beast: Crs and icans after car T-cell therapy for all. Bone Marrow Transplant (2021) 56(3):552–66. doi: 10.1038/s41409-020-01134-4 PMC8592274 33230186

[3] Neelapu SS Locke FL Bartlett NL Lekakis LJ Miklos DB Jacobson CA. Axicabtagene ciloleucel car T-cell therapy in refractory Large b-cell lymphoma. N Engl J Med (2017) 377(26):2531–44. doi: 10.1056/NEJMoa1707447 PMC5882485 29226797

[4] Fischer JW Bhattarai N. Car-T cell therapy: Mechanism, management, and mitigation of inflammatory toxicities. Front Immunol (2021) 12:693016. doi: 10.3389/fimmu.2021.693016 34220853 PMC8250150

[5] Lee DW Santomasso BD Locke FL Ghobadi A Turtle CJ Brudno JN. Astct consensus grading for cytokine release syndrome and neurologic toxicity associated with immune effector cells. Biol Blood Marrow Transplant (2019) 25(4):625–38. doi: 10.1016/j.bbmt.2018.12.758 PMC12180426 30592986

[6] Fowler NH Dickinson M Ghosh M Chen AI Andreadis C Tiwari R. Assessment of healthcare resource utilization and hospitalization costs in patients with relapsed or refractory follicular lymphoma undergoing car-T cell therapy with tisagenlecleucel: Results from the elara study. Transplant Cell Ther (2022) S2666-6367(22):01660–8. doi: 10.1016/j.jtct.2022.09.022 36182104

[7] Johnson PC Jacobson C Yi A Saucier A Dhawale TM Nelson A. Healthcare utilization and end-of-Life outcomes in patients receiving car T-cell therapy. J Natl Compr Canc Netw (2021) 19(8):928–34. doi: 10.6004/jnccn.2020.7678 PMC11221604 33706257

[8] Kenzik KM Johnson PC Bhatia R Bhatia S. Assessment of hospitalizations and emergency department visits after chimeric antigen receptor T-cell therapy among commercially insured patients. JAMA Oncol (2022) 8(7):1068–70. doi: 10.1001/jamaoncol.2022.1044 PMC9073656 35511173

[9] Chaftari P Lipe DN Wattana MK Qdaisat A Krishnamani PP Thomas J. Outcomes of patients placed in an emergency department observation unit of a comprehensive cancer center. JCO Oncol Pract (2022) 18(4):e574–e85. doi: 10.1200/OP.21.00478 PMC9014449 34905410

[10] Porter D Frey N Wood PA Weng Y Grupp SA. Grading of cytokine release syndrome associated with the car T cell therapy tisagenlecleucel. J Hematol Oncol (2018) 11(1):35. doi: 10.1186/s13045-018-0571-y 29499750 PMC5833070

[11] Porter DL Levine BL Kalos M Bagg A June CH. Chimeric antigen receptor-modified T cells in chronic lymphoid leukemia. N Engl J Med (2011) 365(8):725–33. doi: 10.1056/NEJMoa1103849 PMC3387277 21830940

[12] Lipe DN Bourenane SS Wattana MK Gaeta S Chaftari P Cruz Carreras MT. A modified emergency severity index level is associated with outcomes in cancer patients with covid-19. Am J Emerg Med (2022) 54:111–6. doi: 10.1016/j.ajem.2022.02.002 PMC8817422 35152119

[13] Revisions to the standards for the classification of federal data on race and ethnicity. (1997, October 30). Office of management and budget. Available at: https://obamawhitehouse.archives.gov/omb/fedreg_1997standards. Accessed on February 15, 2023.

[14] Atrash S Bano K Harrison B Abdallah AO. Car-T treatment for hematological malignancies. J Investig Med (2020) 68(5):956–64. doi: 10.1136/jim-2020-001290 32200355

[15] Han D Xu Z Zhuang Y Ye Z Qian Q. Current progress in car-T cell therapy for hematological malignancies. J Cancer (2021) 12(2):326–34. doi: 10.7150/jca.48976 PMC7738987 33391429

[16] Zhao Z Chen Y Francisco NM Zhang Y Wu M. The application of car-T cell therapy in hematological malignancies: Advantages and challenges. Acta Pharm Sin B (2018) 8(4):539–51. doi: 10.1016/j.apsb.2018.03.001 PMC6090008 30109179

[17] Davis JA Gaffney KJ McGann M Smith D Edwards K Baldino E. Fever characteristics and impact on safety and efficacy of chimeric antigen receptor T-cell therapy. Clin Lymphoma Myeloma Leuk (2022) S2152-2650(22):01689–5. doi: 10.1016/j.clml.2022.09.005 36319568

[18] Busca A Salmanton-Garcia J Corradini P Marchesi F Cabirta A Di Blasi R. Covid-19 and car T cells: A report on current challenges and future directions from the epicovideha survey by eha-idwp. Blood Adv (2022) 6(7):2427–33. doi: 10.1182/bloodadvances.2021005616 PMC8575532 34749396

[19] Azoulay E Castro P Maamar A Metaxa V de Moraes AG Voigt L. Outcomes in patients treated with chimeric antigen receptor T-cell therapy who were admitted to intensive care (Carttas): An international, multicentre, observational cohort study. Lancet Haematol (2021) 8(5):e355–e64. doi: 10.1016/S2352-3026(21)00060-0 33894170

[20] Long B Yoo MJ Brady WJ Holian A Sudhir A Gottlieb M. Chimeric antigen receptor T-cell therapy: An emergency medicine focused review. Am J Emerg Med (2021) 50:369–75. doi: 10.1016/j.ajem.2021.08.042 34461398

[21] Li L Liu J Xu M Yu H Lv C Cao F. Treatment response, survival, safety, and predictive factors to chimeric antigen receptor T cell therapy in Chinese relapsed or refractory b cell acute lymphoblast leukemia patients. Cell Death Dis (2020) 11(3):207. doi: 10.1038/s41419-020-2388-1 32231200 PMC7105502

[22] Barbera L Taylor C Dudgeon D. Why do patients with cancer visit the emergency department near the end of life? CMAJ (2010) 182(6):563–8. doi: 10.1503/cmaj.091187 PMC2845683 20231340

[23] John S Pulsipher MA Moskop A Hu Z-H Phillips CL Hall EM. Real-world outcomes for pediatric and young adult patients with relapsed or refractory (R/R) b-cell acute lymphoblastic leukemia (All) treated with tisagenlecleucel: Update from the center for international blood and marrow transplant research (Cibmtr) registry. Blood (2021) 138(Supplement 1):428–. doi: 10.1182/blood-2021-146393

[24] Vercellino L Di Blasi R Kanoun S Tessoulin B Rossi C D'Aveni-Piney M. Predictive factors of early progression after car T-cell therapy in Relapsed/Refractory diffuse Large b-cell lymphoma. Blood Adv (2020) 4(22):5607–15. doi: 10.1182/bloodadvances.2020003001 PMC7686887 33180899

[25] Cai C Tang D Han Y Shen E Abdihamid O Guo C. A comprehensive analysis of the fatal toxic effects associated with Cd19 car-T cell therapy. Aging (Albany NY) (2020) 12(18):18741–53. doi: 10.18632/aging.104058 PMC7585129 32973124

[26] Anand K Burns E Sano D Pingali SR Westin J Nastoupil LJ. Comprehensive report of anti-Cd19 chimeric antigen receptor T cells (Car-T) associated non-relapse mortality (Cart-nrm) from faers. J Clin Oncol (2019) 37(15_suppl):2540–. doi: 10.1200/JCO.2019.37.15_suppl.2540

