# Supplementary material for: Emergency department use by patients who received chimeric antigen receptor T cell infusion therapy
Source: Front Oncol. 2023 Mar 17;13:1122329. doi: 10.3389/fonc.2023.1122329 (PMC10064130; doi:10.3389/fonc.2023.1122329)
Supplement: Supplementary file 1 [file DataSheet_1.docx]

**SUPPLEMENTARY MATERIAL**

**TABLES**

**Table S1. Characteristics of chimeric antigen receptor T-cell infusion therapy that our study's 168 patients used**

| **Characteristics** | **No. (%)** |
| --- | --- |
| Therapy type |  |
| Axicabtagene ciloleucel | 108 (64.3) |
| Idecabtagene vicleucel | 21 (12.5) |
| Brexucabtagene autoleucel | 19 (11.3) |
| Tisagenlecleucel | 12 (7.1) |
| Lisocabtagene maraleucel | 8 (4.8) |
| Infusion location |  |
| Outpatient | 0 (0) |
| Inpatient | 168 (100) |

**Table S2. Presentation and characteristics of emergency department (ED) visits by cancer patients who had initiated chimeric antigen receptor T-cell infusion therapy in the past 6 months (n = 276), stratified by the timing of the ED visit after initiation of therapy.**

| **Variable** | **ED visit timing** | | | **P*** |
| --- | --- | --- | --- | --- |
|  | **≤14 days** | **15-90 days** | **>90 days** |  |
| Total | 29 | 153 | 94 |  |
| Acuity |  |  |  | 0.614 |
| Urgent | 16 (55.2) | 94 (61.4) | 57 (60.6) |  |
| Emergent | 12 (41.4) | 55 (35.9) | 37 (39.4) |  |
| Less urgent | 1 (3.4) | 3 (2.0) | 0 (0.0) |  |
| Non-urgent | 0 (0.0) | 1 (0.7) | 0 (0.0) |  |
| ED disposition |  |  |  | 0.871 |
| Admit | 20 (69.0) | 95 (62.1) | 54 (57.4) |  |
| Discharge | 6 (20.7) | 36 (23.5) | 26 (27.7) |  |
| Observation | 3 (10.3) | 20 (13.1) | 11 (11.7) |  |
| Others† | 0 (0.0) | 2 (1.3) | 3 (3.2) |  |
| ICU admission |  |  |  | 0.719 |
| No | 27 (93.1) | 146 (95.4) | 89 (94.7) |  |
| Yes | 2 (6.9) | 7 (4.6) | 5 (5.3) |  |
| Died during the ED visit or subsequent hospital admission |  |  |  | 0.332 |
| No | 29 (100.0) | 146 (95.4) | 87 (92.6) |  |
| Yes | 0 (0.0) | 7 (4.6) | 7 (7.4) |  |
| Top presenting complaint |  |  |  |  |
| Fever | 12 (41.4) | 24 (15.7) | 18 (19.1) | 0.006 |
| Fatigue | 4 (13.8) | 18 (11.8) | 3 (3.2) | 0.030 |
| Abnormal lab results | 1 (3.4) | 12 (7.8) | 15 (16.0) | 0.066 |
| Abdominal pain | 2 (6.9) | 6 (3.9) | 10 (10.6) | 0.096 |
| Shortness of breath | 0 (0.0) | 8 (5.2) | 9 (9.6) | 0.150 |
| Altered mental status | 2 (6.9) | 9 (5.9) | 3 (3.2) | 0.567 |
| CRS | 15 (51.7) | 6 (3.9) | 0 (0.0) | <0.001 |
| ICANS | 3 (10.7) | 6 (3.9) | 0 (0.0) | 0.017 |
| Identified infection | 4 (13.8) | 43 (28.1) | 34 (36.2) | 0.061 |

Abbreviations: CRS, cytokine release syndrome; ICANS, immune effector cell-associated neurotoxicity syndrome.

*P values shown were determined by the Fisher exact test, except for fever as a presenting complaint and identified infection, which was determined by the chi-square test.

**†**Includes visits in which the patient left without being seen, was transferred, or left against medical advice.

**Table S3. Presentation and characteristics of emergency department (ED) visits by cancer patients who had initiated chimeric antigen receptor T-cell infusion therapy in the past 6 months (n = 276), stratified by chimeric antigen receptor T-cell product type.**

| **Variable** | **Chimeric antigen receptor T-cell product type** | | | | |
| --- | --- | --- | --- | --- | --- |
|  | **Axicabtagene ciloleucel** | **Idecabtagene vicleucel** | **Brexucabtagene autoleucel** | **Tisagenlecleucel** | **Lisocabtagene maraleucel** |
| Total visits | 179 | 33 | 26 | 23 | 15 |
| Acuity |  |  |  |  |  |
| Urgent | 106 (59.2) | 22 (66.7) | 13 (50.0) | 15 (65.2) | 11 (73.3) |
| Emergent | 71 (39.7) | 9 (27.3) | 12 (46.2) | 8 (34.8) | 4 (26.7) |
| Less urgent | 2 (1.1) | 1 (3.0) | 1 (3.8) | 0 (0.0) | 0 (0.0) |
| Non-urgent | 0 (0.0) | 1 (3.0) | 0 (0.0) | 0 (0.0) | 0 (0.0) |
| ED disposition |  |  |  |  |  |
| Admit | 114 (63.7) | 16 (48.5) | 13 (50.0) | 17 (73.9) | 9 (60.0) |
| Discharge | 42 (23.5) | 10 (30.3) | 8 (30.8) | 4 (17.4) | 4 (26.7) |
| Observation | 19 (10.6) | 7 (21.2) | 4 (15.4) | 2 (8.7) | 2 (13.3) |
| Others† | 4 (2.2) | 0 (0.0) | 1 (3.8) | 0 (0.0) | 0 (0.0) |
| ICU admission |  |  |  |  |  |
| No | 170 (95.0) | 32 (97.0) | 23 (88.5) | 22 (95.7) | 15 (100.0) |
| Yes | 9 (5.0) | 1 (3.0) | 3 (11.5) | 1 (4.3) | 0 (0.0) |
| Died during the ED visit or subsequent hospital admission |  |  |  |  |  |
| No | 169 (94.4) | 31 (93.9) | 26 (100.0) | 21 (91.3) | 15 (100.0) |
| Yes | 10 (5.6) | 2 (6.1) | 0 (0.0) | 2 (8.7) | 0 (0.0) |
| Top presenting complaint |  |  |  |  |  |
| Fever | 38 (21.2) | 3 (9.1) | 4 (15.4) | 5 (21.7) | 4 (26.7) |
| Fatigue | 16 (8.9) | 4 (12.1) | 2 (7.7) | 3 (13.0) | 0 (0.0) |
| Abnormal lab results | 15 (8.4) | 2 (6.1) | 4 (15.4) | 1 (4.3) | 6 (40.0) |
| Abdominal pain | 10 (5.6) | 2 (6.1) | 3 (11.5) | 2 (8.7) | 1 (6.7) |
| Shortness of breath | 12 (6.7) | 3 (9.1) | 1 (3.8) | 1 (4.3) | 0 (0.0) |
| Altered mental status | 8 (4.5) | 2 (6.1) | 0 (0.0) | 2 (8.7) | 2 (13.3) |

**†**Includes visits in which the patient left without being seen, was transferred, or left against medical advice.

**Table S4. Presentation and characteristics of emergency department (ED) visits by cancer patients who had initiated chimeric antigen receptor T-cell infusion therapy in the past 6 months (n = 276), stratified by underlying cancer type.**

| **Variable** | **Cancer type** | | |
| --- | --- | --- | --- |
|  | **Lymphoma** | **Multiple myeloma** | **Leukemia** |
| Total visits | 225 | 33 | 18 |
| Acuity |  |  |  |
| Urgent | 134 (59.6) | 22 (6.7) | 11 (61.1) |
| Emergent | 88 (39.1) | 9 (27.3) | 7 (38.9) |
| Less urgent | 3 (1.3) | 1 (3.0) | 0 (0.0) |
| Non-urgent | 0 (0.0) | 1 (3.0) | 0 (0.0) |
| ED disposition |  |  |  |
| Admit | 141 (62.7) | 16 (48.5) | 12 (66.7) |
| Discharge | 54 (24.0) | 10 (30.3) | 4 (22.2) |
| Observation | 25 (11.1) | 7 (21.2) | 2 (11.1) |
| Others† | 5 (2.2) | 0 (0.0) | 0 (0.0) |
| ICU admission |  |  |  |
| No | 214 (95.1) | 32 (97.0) | 16 (88.9) |
| Yes | 11 (4.9) | 1 (3.0) | 2 (11.1) |
| Died during the ED visit or subsequent hospital admission |  |  |  |
| No | 215 (95.6) | 31 (93.9) | 16 (88.9) |
| Yes | 10 (4.4) | 2 (6.1) | 2 (11.1) |
| Top presenting complaint |  |  |  |
| Fever | 49 (21.8) | 3 (9.1) | 2 (11.1) |
| Fatigue | 19 (8.4) | 4 (12.1) | 2 (11.1) |
| Abnormal lab results | 23 (10.2) | 2 (6.1) | 3 (16.7) |
| Abdominal pain | 14 (6.2) | 2 (6.1) | 2 (11.1) |
| Shortness of breath | 13 (5.8) | 3 (9.1) | 1 (5.6) |
| Altered mental status | 11 (4.9) | 2 (6.1) | 1 (5.6) |

**†**Includes visits in which the patient left without being seen, was transferred, or left against medical advice.

**Table S5. Cause and place of death for patients who had chimeric antigen receptor T-cell infusion and had an emergency department visit during the study period (n = 63)**

| **Cause and place of death** | **No. (%)** |
| --- | --- |
| Cause of death |  |
| Cancer progression | 11 (17.5) |
| Infection/Sepsis | 9 (14.3) |
| Respiratory failure | 6 (9.5) |
| Multiorgan failure | 5 (7.9) |
| CART-related toxicity | 1 (1.6) |
| Other causes | 4 (6.3) |
| Unknown/undocumented | 27 (42.9) |
| Place of death |  |
| Hospital | 27 (42.9) |
| Home | 14 (22.2) |
| Hospice | 7 (11.1) |
| Unknown/undocumented | 15 (23.8) |

**Table S6. Cause of death for patients who died during the ED visit or subsequent hospital admission (n = 14)**

| **Cause of death** | **No. (%)** |
| --- | --- |
| Infection/Sepsis | 6 (42.9) |
| Cancer progression | 4 (28.6) |
| Multiorgan failure | 2 (14.3) |
| Respiratory failure | 1 (7.1%) |
| CART-related toxicity | 1 (7.1%) |

**FIGURES
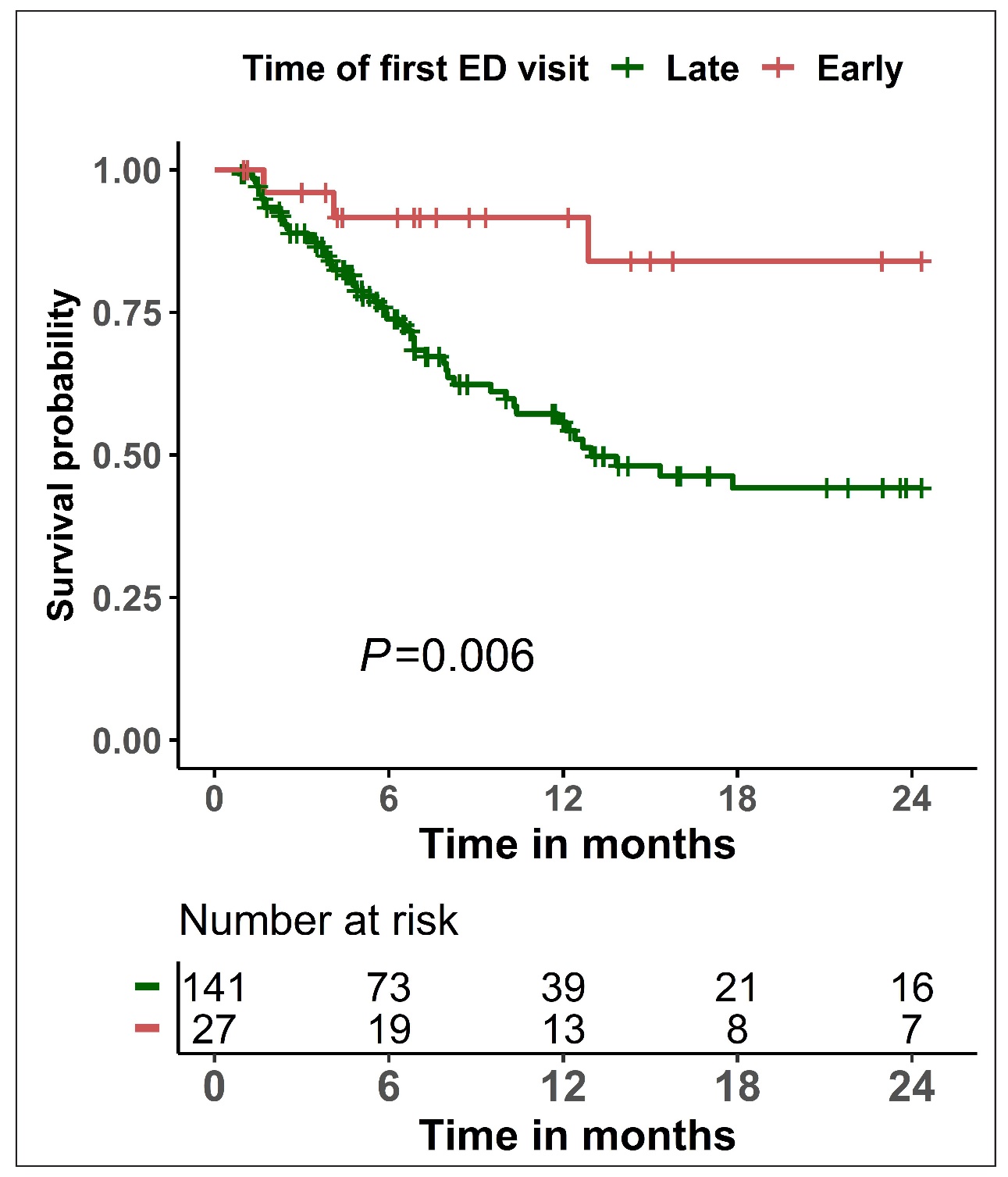
**

**Figure S1. Kaplan-Meier two-year survival curves among patients who visited the emergency department (ED) within 6 months of initiating chimeric antigen receptor T-cell infusion therapy, stratified by timing of the first ED visit (early: ≤14 days; late: >14 days).**
